# The Impact of COVID-19 on Smoking Behaviours and Support for Smoke-Free Zones in Saudi Arabia

**DOI:** 10.3390/ijerph18136927

**Published:** 2021-06-28

**Authors:** Mansour Tobaiqy, Andrew MacLure, Dennis Thomas, Katie MacLure

**Affiliations:** 1 Department of Pharmacology, College of Medicine, University of Jeddah, P.O. Box 45311, Jeddah 21512, Saudi Arabia; 2 Independent Researcher, Aberdeen AB32 6RU, UK; akmaclure@outlook.com; 3 School of Medicine and Public Health, College of Health, Medicine and Wellbeing, University of Newcastle, Callaghan, NSW 2308, Australia; dennis.thomas@newcastle.edu.au; 4 Independent Research Consultant, Aberdeen AB32 6RU, UK; katiemaclure@outlook.com

**Keywords:** smoking cessation, quitting tobacco products, barriers for quitting, secondhand smoking, smoke free zones, COVID-19, Saudi Arabia

## Abstract

This article focuses on the impact of COVID-19 on smoking and smoking cessation behaviours and support for smoke-free zones in Jeddah, Saudi Arabia. A pre-tested structured survey was distributed by email in October–November 2020 to students and staff at the University of Jeddah. Responses were analysed using descriptive statistics with summative content analysis of open text. Participants providing open text comments (*n* = 374/666; 56.4%) were non-smokers (*n* = 293; 78.3%), former smokers (*n* = 26; 7.0%) and current smokers (*n* = 55; 14.7%). Some had household members (*n* = 220; 58.8%) and friends who smoke (*n* = 198; 52.9%) plus daily exposure to secondhand smoke at home (*n* = 125; 33.4%). There was an awareness during COVID-19 of: smoking inside cafes/restaurants and other indoor and outdoor public places; exposure to warnings in the media both against and promoting smoking; widespread support for smoke-free zones. Smokers plans for accessing smoking cessation support are inconsistent with retrospective reports. Many express positivity highlighting reductions in smoking but there were also negative reports of increased smoking. The COVID-19 pandemic has affected every aspect of society worldwide. People have been at home more with restricted freedom of movement and limitations on social liberty. These individual accounts can help to focus evidence-based smoking prevention and cessation programmes during and post-COVID-19.

## 1. Introduction

Globally, the World Health Organization (WHO) engages with countries to adopt policies and strategies which strive to encourage and support people to quit smoking [1,2,3]. This is because of the widely accepted health hazards to the smoker and those around them exposed to secondhand smoke. The WHO Global Action Plan has a stated aim of reducing smoking worldwide by 30% by 2025 [4]. However, to date, only 23 countries were on track before the global COVID-19 pandemic to meet the target [4,5]. Undoubtedly, cigarette smoking is associated with increased risk for the development of cancer and cardiovascular diseases, both of which were the leading causes of death in recent decades [6].

While figures for deaths as a direct consequence of smoking are estimated at 7 million per year, those attributable to secondhand smoking are reported as 1.2 million [4]. While these avoidable deaths are clearly a major issue, mechanisms to tackle smoking and promote smoking cessation have yet to reach the projected targets. In 2015, some areas (Africa, Eastern Mediterranean) were even predicted to have increased numbers of smokers [5].

Many countries have adopted smoke free areas in a push towards reduction in exposure to secondhand smoking in both public and private places [2]. Price rises, plain packaging, advertising bans, increased awareness raising, support for smoking cessation have all been enacted in the WHO campaigns down the decades with varying degrees of success to tackle what is seen as a smoking pandemic [1,2,3,4,5].

In late 2019, COVID-19 began, which the WHO declared as a pandemic in March 2020. At the time of writing (February 2021) there have been over 110 million cases worldwide with more than 2.4 million deaths attributed to COVID-19 [7]. Initial reports in early 2020 made an association between smoking and COVID-19. Firstly, that smoking might be a protective factor against infection [8]. Secondly, that smokers who did contract the virus would be more severely affected [9].

In March 2020, Varadavas and Nikitara (2020) published one of the first systematic reviews focused on COVID-19 and smoking [9]. Their findings related to severity of the disease amongst current and former smokers that was based on only five studies, all of which were from mainland China where the outbreak began [8]. The Centre for Disease Control and Prevention (CDC) advised caution on early indications related to smoking noting the lack of early data [9]. However, smoking, along with other underlying health conditions, was noted as a risk factor for respiratory infections [9]. A flurry of articles and statements during 2020 were inconclusive with some supporting the second claim around smoking and severity of COVID-19 [10]. Some articles took care to discourage people from taking up smoking, or engaging in harmful ‘self-destructive behaviours,’ while down-playing the limited evidence to support the first claim of smoking acting as a protective factor against COVID-19 infection [8,11,12,13]. A more measured response from van Westen-Lagerweij et al., (2021) has been published which debunks the COVID-19 smoking myths (protective factor, more severely affected) further reinforcing the anti-smoking campaigns [14].

Hefler and Gartner (2020) suggested the social restrictions of the pandemic presented an opportunity for smokers to quit and for cigarettes to be removed from general retail sale [15]. Clearly, a well-supported view [1,2,4]. However, there has been evidence that the ‘stay home, save lives’ restrictions during COVID-19 lockdowns have increased household exposure to secondhand smoking [16,17,18,19]. As time has gone on and the world strives to vaccinate against COVID-19 and its variants, a timely follow-up study in Turkey reported increased smoking cessation during the pandemic of 31.1% up from the 1 year follow up of 23.7% [20]. Studies from other countries have reported mixed results around smoking cessation during the pandemic [21,22,23].

In Saudi Arabia, where smoking is associated with an estimated 70,000 deaths per year, many preventative and protective measures have been adopted [24]. Smoking in many public and enclosed areas has been banned in Saudi Arabia since 2012 with more stringent policies invoked in line with WHO Framework Convention on Tobacco Control (FCTC) in recent years [2,25,26]. Support for smoking cessation has seen additional healthcare clinics and mobile smoking cessation units open [24]. Like many Middle Eastern countries, Saudi Arabia banned the use of waterpipes in cafes and restaurants during the COVID-19 pandemic amid fears around spread of the disease. However, despite these measures and increased support for smoking prohibitions, tobacco smoking habits and the use of waterpipes has likely moved to home settings. There is evidence that younger people are still taking up smoking despite the education programmes and anti-tobacco campaigns in place [27].

Our recent systematic review found the literature on smoking in Saudi Arabia to be extensive [27]. However, it noted the attitudes of both smokers and non-smokers towards smoking cessation needed further exploration. On that basis, the systematic review asked the question, ‘What are the attitudes of smokers and non-smokers towards smoking cessation in Saudi Arabia?’ Findings from the systematic review noted the paucity of quality evidence on which to base any recommendations and that the smoking pandemic was still resonant in Saudi Arabia. Additionally, it raised the issue of research not keeping pace which is particularly pertinent given the COVID-19 pandemic [27]. Therefore, despite strong Ministry of Health support for education programs that try to prevent the uptake of smoking, policy-driven action to reduce environmental second-hand smoking, and provision of support for smoking cessation, more needed to be done with further research undertaken to evaluate outcomes [27]. Following on from the systematic review, a survey was conducted resulting in two articles. The first reported the experiences, knowledge and attitudes of a university population toward smoking cessation. The aim of this second article was to focus on the impact of COVID-19 on smoking behaviours and support for smoke-free zones in Jeddah, KSA.

## 2. Materials and Methods

The survey content was based on a systematic review which identified validated and non-validated tools as well as shortcomings of previous studies and recommendations [27]. The Google sheets based online survey was first developed in English (the language of the WHO, CDC and Challenges to Stopping Smoking [CSS-21] validated tools) [1,2,3,4,10,28]. It consisted of 29 top level questions several of which were subdivided or in matrix format. Nine of these gathered demographic data including: sex, age, marital status, nationality, student or university staff, household income, smokers in the household and amongst friends, their own smoking status (never smoked, former smoker (last 12 months), former smoker (more than 12 months) and smoker). It was reviewed for face and content validity using the ‘Think Aloud’ approach, in which people are asked to verbalise their thought processes while completing the survey, with smoking and non-smoking academics (smoker *n* = 1; former smoker *n* = 1; never smoked *n* = 2), students *n* = 2 and members of the general public *n* = 3). Amendments were made before translation and back translation in Arabic with further face and content validity conducted to confirm the accuracy of the translation (two Arabic speaking postgraduate students fluent in English). Both languages were made available to the respondents via an online link in an email. This was distributed in October and November 2020 to students and staff at the University of Jeddah explaining the purpose of the survey, the voluntary nature of completion and that there were no mandatory questions.

For context, at the time of conducting the survey, mask wearing in public was mandatory and mass gatherings banned. Since March 2020, students and staff were meant to be off campus, limited businesses were open; people were instructed to stay at home only going out for essential work and travel [29].

Aspects of the survey reported in this article are: demographics and secondhand smoking at home, for all who responded to the survey, and, for those providing additional open text comments on the impact of COVID-19 on smoking behaviours, their awareness of smoking at different venues, exposure in the media and, finally, support for smoke-free zones. A copy of the survey is provided in the Supplementary Material. Responses were downloaded to and analysed using descriptive statistics in opensource JASP Team (2020). JASP (Version 0.14.1) [Computer software]. Summative content analysis of open text responses looking for similarities, patterns and differences was conducted in MS Word with discussion amongst authors to form consensus on which comments were positively, negatively and neutrally worded [30].

The study was reviewed by the University of Jeddah Bioethical Committee of Scientific and Medical Research Ethical Review Board (3 April 2020; UJ-REC-002) and was funded by an International Corporation Program Grant from the Ministry of Education, University of Jeddah.

## 3. Results

A total of 666 responses, predominantly reported elsewhere, were collected by the survey. The survey link had been emailed to 34,872 university members (2500 faculty member, 1557 technician and administrative staff, and 30,815 students) giving an overall response rate of 1.9%.

Respondents, as per Table 1, are presented in terms of two groups: those who answered the open text question on the impact of COVID-19 on smoking behaviours, and those who did not comment. Overall, respondents were mainly female (*n* = 417; 66.2%), Saudi nationals (*n* = 623; 93.5%), aged 25 years old and under (*n* = 501; 75.2%) and students (*n* = 541; 82.1%). Most were non-smokers (*n* = 556; 83.5%). In terms of secondhand smoking, more than half had household members who smoke (*n* = 394; 59.2%) with just under half having friends who smoke (*n* = 321; 48.2%).

Additionally, in Table 1, for more than half of the respondents, ‘smoking is never acceptable inside of your home’ (*n* = 391; 58.6%) and ‘never’ practiced (*n* = 375; 56.6%). However, there were ‘exceptions’ made (*n* = 131; 19.7%) with smoking taking place ‘daily’ in some homes (*n* = 206; 31.0%).

The responses of those who later provided open text comments (Did comment *n* = 374; 56.2%) were largely similar to those who did not provide comments (Did not comment *n* = 292 (43.8%) on the impact of COVID-19 on smoking behaviours. There were slight differences between the two groups. Those who provided comment were more likely to be male ((*n* = 155 (23.3%); *n* = 92 (13.8%)), Saudi nationals ((*n* = 347 (52.1%); *n* = 276 (41.4%)) and have friends who smoke ((*n* = 198 (29.7%); *n* = 123 (18.5%)). On this basis of demographically similar groups, the focus of this paper and further results relate only to those participants providing open text comments.

Participants were asked if they had been aware of people smoking in a range of commonly visited places over the last 30 days. The results in Table 2 provide evidence that smoking was still widely visible, even during the COVID-19 pandemic, particularly ‘inside cafes and restaurants’ (*n* = 205; 54.8%) and ‘outside on university campus’ (*n* = 173; 45.5%). There were high levels of support for smoking prohibition ranging from 70.9% to 93.6% in contained and indoor spaces. 

Otherwise, sporting events or venues were the most remembered as showing warnings against smoking (*n* = 264; 70.6%) with billboards or posters, social media and cigarette packaging each reported by over 60%. Respondents reported seeing promotional material for smoking and smokeless products in stores where the products were sold (39.8%; 21.1%) but less so on social media (30%; 26.7%) and on the internet (24.6%; 23.5), respectively.

Awareness of smoking related warnings and promotional material, presented in Table 3, in the media varied greatly. Warnings against smoking during the pandemic were least noted in the cinema (*n* = 127; 34%); however, many venues may have been closed due to COVID-19 or participants may not have visited a cinema in the previous 30 days. 

Participants were asked to comment on whether their attitude towards smoking had been impacted by the COVID-19 pandemic. Table 4 has example quotes from non-smokers (*n* = 293; 78.3%), former smokers (*n* = 26; 7.0%) and current smokers (*n* = 55; 14.7%), which have each been grouped and tallied, in order, as positive, neutral and negative comments. The tally count in summative content analysis is a blunt instrument but it does give an impression of the overall positivity, neutrality and negativity of the three smoking groups [30]. While smokers were fairly evenly spread in their comments (positive *n* = 20; neutral *n* = 19; negative *n* = 17), respectively, those who identified as former smokers were more positive (*n* = 16; *n* = 4; *n* = 6) and non-smokers less negative (*n* = 115; *n* = 117; *n* = 62) overall.

Those who had managed to stop smoking for 21 days or more (*n* = 29) relied on a smoking cessation clinic (*n* = 2), nicotine replacement therapy (*n* = 3), switch to smokeless (*n* = 2), low tar alternatives (*n* = 6), other medicines (*n* = 1) but many just stopped using their will power (*n* = 8) while the remainder did not specify any smoking cessation support mechanisms (*n* = 9). For those who had quit for the shorter period of 20 days or less (*n* = 20), more relied on smoking cessation clinic (*n* = 6), low tar alternatives (*n* = 7), other medicines (*n* = 3) nicotine replacement therapy (*n* = 2), switch to smokeless (*n* = 1), but, again, will power was an option (*n* = 3) while the remainder did not specify any smoking cessation support mechanisms (*n* = 7). Those expressing an intention to quit within the next 12 months (*n* = 35) gave a preference for face-to-face counselling (*n* = 14), access to an on campus mobile smoking cessation unit (*n* = 10), telephone counselling (*n* = 12), supportive text messages (*n* = 9), mobile phone app (*n* = 6), access to online resources (*n* = 2), medication options including NRT (*n* = 9), support group face-to-face (*n* = 0), support group online (*n* = 5) or alternative treatments such as hypnotherapy or acupuncture (*n* = 8).

## 4. Discussion

Conducted during the COVID-19 pandemic, this survey provides novel insight into smokers, non-smokers and former smokers views on smoking behaviours and smoke-free zones. Levels of smoking reported in the study are similar to the overall statistics for Saudi Arabia [1,2,4,6].

While the majority of participants are non-smokers, they are exposed to secondhand smoking in the home. As reported elsewhere, this has been particularly challenging to avoid during the COVID-19 restrictions [16,17,18,19,20,21,22]. It is noticeable that no data is reported in the recent literature, even from the WHO or CDC, for comparison of this study with regard to secondhand smoking. That said, some agree it is likely that secondhand smoking in the home has increased during lockdown, which suggests smokers should be encouraged to at least smoke outside or in well-ventilated areas away from non-smokers [18,19]. Cultural norms have forced some to quit smoking during the pandemic because of lack of access to cigarettes, lack of an area to smoke, or because their smoking was not previously known to the family and would be met with disapproval [31].

Despite international and national campaigns described earlier, there remains a perception that smoking is still prevalent in the community and even promoted in many areas of daily living [1,2,24,25,26]. The response rate is too low to claim broad support for smoke-free zones amongst the University of Jeddah staff and students. What we can say is that the responses of those who took part did align well with the WHO FCTC and national anti-smoking campaigns [2]. It is important to note the anomalies: participants indicate being aware of smoking in places where smoking is banned, not only during COVID-19, but also as part of national policy; participants also report being exposed not only to warnings against smoking but also promotion of both tobacco and smokeless options. This awareness of smoking was reported when some of these public gathering places, including campuses, were closed due to the pandemic. However, these are the perceptions of the participants so findings from this study may help focus smoking prevention and cessation activities provided they take into account the vagaries and nuances of public perceptions; listen to what they say but also see what people do suggests following up this survey with observational research to help shape the secondhand smoking policy response. This and other studies show broad support for Saudi Arabia’s WHO commitments and targets in seeking to reduce both smoking and secondhand smoking [4,6,25]. However, given the disparities reported here between prospective approaches to smoking cessation and retrospective identification of what actually worked, review of the strategies on offer may be beneficial underpinned by further research focused on the ‘reality on the ground’.

These are individual voices, personal accounts, of the impact on smoking during the pandemic. Many express positivity highlighting reductions in smoking but there are also negative reports of increased smoking during the pandemic, contrary to best advice [14]. Those already strongly against smoking report unchanged or strengthened opposition. Many mention being more exposed to smoking within the home than they were when outside with friends pre-COVID-19 [1,2,4,20,21,22,23]. It is too early at this point to provide accurate figures on changes in secondhand smoking but some studies are laying the groundwork [16,17,18,19]. Some expressed shock that people would continue to smoke despite indications that, as a respiratory infection, they might be more severely affected if they contracted COVID-19 [9,14].

The study is not without its limitations, first and foremost the low response rate. Also. it was conducted in only one university and completed by relatively few staff and students. The content analysis on open text responses was conducted through discussion amongst the multidisciplinary authoring team but it did still rely on our interpretation. However, participants’ voices which are quoted verbatim are nevertheless valid and give valuable insight into the level of support for smoke-free zones which should be helpful in targeting future smoking prevention and cessation programmes.

## 5. Conclusions

The COVID-19 pandemic has affected every aspect of society worldwide. People have been at home more with restricted freedom of movement and limitations on social liberty. Smoking behaviours have been impacted for the better for some and for the worse for others. These individual accounts can help to focus evidence-based smoking prevention and cessation programmes during and post-COVID-19 in Saudi Arabia as well as supporting further smoke-free zones.

## Figures and Tables

**Table 1:** Demographics of respondents who did and did not comment on the impact of COVID-19 on smoking (***N*** = 666).

Characteristic		Did Comment *N* = 374 (56.2%)	Did Not Comment *N* = 292 (43.8%)
Gender	Male	155 (23.3)	92 (13.8)
	Female	219 (32.9)	198 (29.7)
	Missing data	0 (0)	2 (0.3)
Nationality	Saudi	347 (52.1)	276 (41.4)
	Non-Saudi	25 (3.8)	14 (2.1)
	Missing data	2 (0.3)	2 (0.3)
Age	25 years old or less	272 (40.8)	229 (34.4)
	26–50 years old	92 (13.8)	56 (8.4)
	51 years of age or older	10 (1.5)	7 (1.1)
University role	Students	292 (43.8)	249 (37.4)
	* Staff	76 (11.4)	46 (6.9)
	Missing data	2 (0.3)	1 (0.2)
Smoking status	Never smoked	293 (44.0)	263 (39.5)
	Smoker	55 (8.3)	17 (2.6)
	Former smoker	26 (3.9)	12 (1.8)
Household members who smoke	Yes	220 (33.0)	174 (26.1)
	No	154 (23.1)	114 (17.1)
	Missing data	0 (0)	4 (0.6)
Friends who smoke	Yes	198 (29.7)	123 (18.5)
	No	176 (26.4)	168 (25.2)
	Missing data	0 (0)	1 (0.2)
Smoking rules at home	Smoking is acceptable inside your family home	31 (4.7)	18 (2.7)
	There are no rules about smoking in your home	54 (8.1)	39 (5.9)
	Smoking is generally not acceptable inside your home but there are exceptions	80 (12.0)	51 (7.7)
	Smoking is never acceptable inside of your home	209 (31.3)	182 (27.3)
	Missing data	0 (0)	2 (0.3)
Frequency of smoking inside the home	Daily	125 (18.8)	81 (12.2)
	Weekly	13 (2.0)	6 (0.9)
	Monthly	8 (1.2)	6 (0.9)
	Less often than monthly	23 (3.5)	20 (3.0)
	Never	203 (30.5)	174 (26.1)
	Missing data	2 (0.3)	5 (0.7)

* Given the low number of respondents, ‘staff’ are grouped from: Academic staff (*n* = 85; 12.7%); Administrative staff (*n* = 26; 4.0%); Technicians (*n* = 7; 1.1%); Health Centre staff (*n* = 4; 0.6%).

**Table 2:** Awareness of smoking in the past 30 days at a range of places (*n* = 374).

Places *n* (%)	Awareness of Smoking	Support for Smoke-Free Zones		
		Yes	No	Do Not Know
In private transport	149 (39.8)	265 (70.9)	74 (19.8)	31 (8.3)
On public transport	162 (43.3)	337 (90.1)	21 (5.6)	14 (3.7)
Inside restaurants or cafes	205 (54.8)	273 (73.0)	66 (17.6)	33 (8.8)
Inside a cinema, theatre, music sport or similar venue/event	48 (12.8)	334 (89.3)	24 (6.4)	11 (2.9)
Outside a cinema, theatre, music sport or similar venue/event	116 (31.3)	202 (54.0)	117 (31.3)	49 (13.1)
Outdoors on university campus	173 (45.5)	226 (60.4)	99 (26.5)	42 (11.2)
Indoors on university campus	99 (26.5)	347 (92.8)	18 (4.8)	9 (2.4)
Inside government buildings	59 (15.8)	350 (93.6)	15 (4.0)	8 (2.1)
Inside healthcare facilities	12 (3.2)	350 (93.6)	17 (4.5)	5 (1.3)

**Table 3:** Exposure to smoking in the media in the last 30 days (*n*= 374) with multiple responses allowed.

Media *n* (%)	Warning of Dangers of Use or Encouraging Quitting of Tobacco Products	Promoting Tobacco Smoking Products	Promoting Smokeless Products
Newspapers or magazines (printed copies)	202 (54.0)	57 (15.2)	41 (11.0)
Television	215 (57.5)	44 (11.8)	56 (15.0)
Radio	169 (45.2)	19 (5.1)	34 (9.1)
Billboards or posters	225 (60.2)	68 (18.2)	53 (14.2)
In stores where tobacco products are sold	194 (51.9)	149 (39.8)	79 (21.1)
Cinemas	127 (34.0)	37 (9.9)	38 (10.2)
Public transportation vehicles or stations	160 (42.8)	26 (7.0)	30 (8.0)
Sporting events or venues	264 (70.6)	27 (7.2)	30 (8.0)
Internet (non-social media)	207 (55.3)	92 (24.6)	88 (23.5)
Social media	228 (61.0)	112 (30.0)	100 (26.7)
Cigarette packaging	229 (61.2)	91 (24.3)	45 (12.0)

**Table 4:** Example quotes of the impact of COVID-19 on smoking from non-smokers, former smokers and smokers (*n* = 374).

Smoking Status	Comments		
	Positive	Neutral	Negative
Non-smokers (*n* = 294)	*n* = 115 ‘I think that smoking has decreased because of the quarantine and their inability to buy cigarettes, and for me, my father was previously a very heavy smoker and now he uses the vape and gradually reduces the amount of nicotine until he no longer uses it and this shift was during the quarantine period’ ‘I now avoid places where smoking is permitted, and I also avoid meeting people who smoke’ ‘I no longer think to experiment with smoking’ ‘The Corona pandemic, despite its trauma, has positively affected smokers, as I do not notice anyone who smokes in restaurants, malls or the street very little’ ‘It affected positively as many people quit smoking due to the risks it poses to the lung, as well as smokers are considered more vulnerable to corona virus and less likely to cure’ ‘I know a lot of people who are social smokers, especially with smoking Hookah. I think the quarantine helped (some) people lessen their social smoking habits. And I think for Females in Saudi Arabia, where it is less socially acceptable for them to smoke, spending more time at home meant that they smoked a lot less because their family would not like that’	*n* = 117 ‘It never affected me and I consider smoking harmful, a bad habit and completely uncivilized’ ‘I do not agree to smoking before and after the pandemic and I reject it completely’ ‘It didn’t affect me because I’m a non-smoker’ ‘It did not affect me because I do not smoke, thank God’ ‘Praise be to Allah, I do not smoke, neither before nor after’ ‘It didn’t make a difference because I hadn’t tried smoking before’ ‘I am not affected because I do not smoke’	*n* = 62 ‘In my opinion, this habit was made worse by the quarantine and isolation at home’ ‘I think the pandemic has increased the rate of smoking because everyone is sitting in their homes’ ‘The Corona pandemic obligated people to stay more in their homes, which put them under pressure of isolation and family conflict. This led to an increase in smoking habits among smokers to relieve psychological pressure, which in turn negatively affected the rest of the non-smoking individuals and also accelerated the harm to smokers’ ‘My exposure to secondhand smoke increased because of the presence of smokers in the home with me’ ‘Certainly, my position has become more negative about smoking because it has the greatest impact on destroying the respiratory system, and of course Corona is a respiratory virus, which will have more effect on smokers in particular’
Former smokers (*n* = 26)	*n* = 16 ‘It was very influential, smoking is greatly affected by the psychological state of the person, but I was able to overcome this feeling and be able to complete not smoking for 3 years now, praise be to Allah’ ‘I now avoid places where smoking is permitted, and I also avoid meeting people who smoke’ ‘It affected me positively, and it is one of the reasons for my quitting smoking because smoking is forbidden in the house and the reasons for home quarantine and not leaving the house and seeing friends are one of the reasons for my quitting smoking’	*n* = 4 ‘I wasn’t affected, as I have an inner conviction, and I was convinced that I would quit smoking’ ‘COVID didn’t change my smoking habits’	*n* = 6 ‘I don’t smoke but all the smokers that I know smoked excessively’ ‘It was the only outlet for smokers and they had nothing to do and were also unable to go out, so they rushed to smoke’
Smokers (*n* = 56)	*n* = 20 ‘I tried very hard to stop smoking for the reason that the complications of Corona are strong for the smoker, and this is one of the reasons that will make me stop smoking soon, God willing’ ‘During the ban period I was bingeing but after a while my smoking more than halved’ ‘It was one of the reasons for my quitting smoking’ ‘Positive saving money and reducing smoking at least’	*n* = 19 ‘My smoking habits were social in practicing them and getting them so the restrictions COVID-19 caused made me stop smoking (it wasn’t an option). But once there was less restrictions I started smoking again, but I quit for 6 months +’ ‘I smoked regular cigarettes more, then I switched to electronic hookahs, and stopped regular cigarettes’ ‘None. I can stop smoking and go back whenever I want. I have tremendous control’	*n* = 17 ‘I started smoking during this period’ ‘My smoking increased during the quarantine period due to boredom and lack of entertainment’ ‘It did not affect me and I do not intend to quit’ ‘Negatively, the interruption of smoke due to corona, was the reason for stopping smoking for a period, and the fear that smokers are more susceptible to the virus’

## Data Availability

All data are reported within the study.

## Supplementary Materials

The following are available online at https://www.mdpi.com/article/10.3390/ijerph18136927/s1, Final survey.
