# Supplementary material for: The Impact of COVID-19 on Smoking Behaviours and Support for Smoke-Free Zones in Saudi Arabia
Source: Int J Environ Res Public Health. 2021 Jun 28;18(13):6927. doi: 10.3390/ijerph18136927 (PMC8297188; doi:10.3390/ijerph18136927)
Supplement: Supplementary file 1 [file ijerph-18-06927-s001.zip › ijerph-1242978-supplementary.pdf]

Experiences, knowledge and attitudes of University of Jeddah's Students and Staff toward smoking cessation

Mansour Tobaiqy, Johnson George, Dennis Thomas, Andrew MacLure, Katie MacLure

Saudi Arabia's National Transforming Program (2020) highlights the need to improve health with a great focus on smoking cessation programs. The Law in Saudi Arabia defines smoking as 'the use of tobacco and its products, such as, cigarettes, cigars, tobacco leaves, tobacco molasses or any other product containing tobacco, either through cigarettes, cigars, pipe, snus, hookah, or chewing tobacco, or any other form.' This was updated in 2018 to include e-cigarettes and e-hookah.

WHETHER YOU ARE A SMOKER OR NOT, we are interested in capturing your experiences, knowledge and attitudes towards smoking cessation and would greatly appreciate your participation in this survey. You are under no obligation to take part and can choose to skip any questions you don't want to answer. We do not collect any identifiable information about you in the survey, so your anonymity is protected. The survey is available in both Arabic and English by following the appropriate link. You will receive one reminder email but please only complete the survey once. If you do decide to take part, there is a follow on link after completion which enables you to claim 10 volunteer hours.

This study has been reviewed by the University of Jeddah Ethical Review Board and is funded by an International Corporation Program Grant from the Ministry of Education, University of Jeddah. Assistant Professor Dr Mansour Tobaiqy leads this international research team with members from the United Kingdom and Australia.

If you have any questions about the survey, please contact:

Dr Mansour Tobaiqy mtobaiqy@uj.edu.sa
Assistant Professor
Pharmacology Department
School of Medicine
University of Jeddah

Section A - Background Characteristics

A00. Firstly, some questions about you and your background

1. A01. Gender

Mark only one oval.

- Male
- Female

2. A02. Age in years

Mark only one oval.

- 15 or younger
- 16-20
- 21-25
- 26-30
- 31-35
- 36-40
- 41-45
- 46-50
- 51 or older

3. A03. Marital status

Mark only one oval.

- Single
- Married
- Divorced / Widowed

4. A04. Nationality

Mark only one oval.

- Saudi
- Non-Saudi

5. A05. Which best describes your status with the university

Mark only one oval.

- Undergraduate Student
- Postgraduate Student
- Technician
- Health Centre Staff
- Administrative Staff
- Academic Staff
- Other: ____________________

6. A06. What is your total monthly household income

Mark only one oval.

- Less than 5000 SAR
- 5000 - 9999
- 10000 - 14999
- 15000 - 19999
- 20000 - 24999
- 25000 SAR or more

7. A07. Which members of your household are smokers (choose all that apply)

Tick all that apply.

- [ ] Grandparents
- [ ] Parents
- [ ] Uncles and Aunts
- [ ] Spouse
- [ ] Brothers and Sisters
- [ ] Cousins
- [ ] Children
- [ ] Staff
- [ ] None of the above

8. A08. What proportion of your friends, that you spend time with on a regular basis, are smokers

Mark only one oval.

- ( ) All
- ( ) Most
- ( ) Half
- ( ) A few
- ( ) Only one or two
- ( ) None

9. A09. Which of the following best describes your smoking status

Mark only one oval.

- ( ) Never smoked *Skip to question 23*
- ( ) Former smoker - quit within the last 12 months *Skip to question 14*
- ( ) Former smoker - quit more than 12 months ago *Skip to question 23*
- ( ) Smoker *Skip to question 10*
- ( ) Other: ______________________

Section B - Smoking

B00. I would now like to ask you some questions about smoking. That's tobacco, for example, cigarettes, cigars, pipes, also smokeless products including waterpipe and electronic cigarettes

10. B01. How old were you when you first started smoking

Mark only one oval.

- Under 15 years
- 15-19
- 20-24
- 25 years or older

11. B02. Current smoking methods and frequency of use

Mark only one oval per row.

	Daily	Weekly	Less often	Never
Factory made cigarettes	⬭	⬭	⬭	⬭
Roll your own cigarettes with filters	⬭	⬭	⬭	⬭
Roll your own cigarettes without filters	⬭	⬭	⬭	⬭
Pipes full of tobacco	⬭	⬭	⬭	⬭
Cigars, cheroots, cigarillos	⬭	⬭	⬭	⬭
Waterpipe	⬭	⬭	⬭	⬭
Electronic cigarettes	⬭	⬭	⬭	⬭
Chewing tobacco	⬭	⬭	⬭	⬭
Electronic hookah	⬭	⬭	⬭	⬭

12. B03. How soon after you wake up do you usually have your first smoke

Mark only one oval.

- Within 5 minutes
- 6 - 30 minutes
- 31 - 60 minutes
- More than 60 minutes

13. B04. On average, how much do you spend on smoking each week

Mark only one oval.

- Less than 50 SAR
- 50 - 99
- 100 - 149
- 150 - 199
- 200 - 249
- 250 - 299
- 300 - 349
- 350 - 399
- 400 - 449
- 450 - 499
- 500 SAR or more

Section C - Smoking Cessation

C00. The next questions ask about any attempts to stop smoking that you might have made within the past 12 months

14. C01. During the past 12 months, how many times have you tried to stop each type of smoking

Mark only one oval per row.

	1	2	3	4	5	Never tried
Factory made cigarettes						
Roll your own cigarettes with filters						
Roll your own cigarettes without filters						
Pipes full of tobacco						
Cigars, cheroots, cigarillos						
Waterpipe						
Electronic cigarettes						
Chewing tobacco						
Electronic hookah						

15. C02. Again, thinking about the last time you tried to quit during the past 12 months, how long did you stop for each type of smoking

Mark only one oval per row.

	Months	Weeks	Days	Less than 24 hours	Never tried
Factory made cigarettes	◯	◯	◯	◯	◯
Roll your own cigarettes with filters	◯	◯	◯	◯	◯
Roll your own cigarettes without filters	◯	◯	◯	◯	◯
Pipes full of tobacco	◯	◯	◯	◯	◯
Cigars, cheroots, cigarillos	◯	◯	◯	◯	◯
Waterpipe	◯	◯	◯	◯	◯
Electronic cigarettes	◯	◯	◯	◯	◯
Chewing tobacco	◯	◯	◯	◯	◯
Electronic hookah	◯	◯	◯	◯	◯

16. C03. Again, thinking about the last 12 months, what is the longest you've stayed quit for each type of smoking in days

Mark only one oval per row.

	Less than 1 day	1 - 5	6 - 10	11 - 20	21 - 30	31 - 60	More than 60 days	Never tried
Factory made cigarettes	◯	◯	◯	◯	◯	◯	◯	◯
Roll your own cigarettes with filters	◯	◯	◯	◯	◯	◯	◯	◯
Roll your own cigarettes without filters	◯	◯	◯	◯	◯	◯	◯	◯
Pipes full of tobacco	◯	◯	◯	◯	◯	◯	◯	◯
Cigars, cheroots, cigarillos	◯	◯	◯	◯	◯	◯	◯	◯
Waterpipe	◯	◯	◯	◯	◯	◯	◯	◯
Electronic cigarettes	◯	◯	◯	◯	◯	◯	◯	◯
Chewing tobacco	◯	◯	◯	◯	◯	◯	◯	◯
Electronic hookah	◯	◯	◯	◯	◯	◯	◯	◯

17. C04. During the last 12 months, did you use any of the following to try to stop smoking (choose all that apply)

Tick all that apply.

- [ ] Counselling at a smoking cessation clinic
- [ ] The on campus mobile smoking cessation clinic
- [ ] Nicotine replacement therapy such as patch or gum
- [ ] Other prescription medications
- [ ] Traditional medicines
- [ ] Quitline or a smoking telephone support
- [ ] Online support group or smartphone app
- [ ] Switching to smokeless tobacco
- [ ] Switching to light, mild or low tar

Other: [ ] ____________________

18. C05. During any visit to a healthcare provider in the last 12 months, were you (choose all that apply)

Tick all that apply.

- [ ] Asked if you smoked tobacco products
- [ ] Advised to quit smoking tobacco products
- [ ] Asked if you used smokeless products
- [ ] Advised to quit smokeless products
- [ ] Offered advice on quitting
- [ ] Offered an appointment at a smoking cessation clinic

19. C06. Which of the following best describes your thinking about quitting smoking

Mark only one oval.

- ( ) I am currently trying to quit
- ( ) I am planning to quit within the next month
- ( ) I am thinking about quitting within the next 12 months
- ( ) I will quit someday but not within the next 12 months
- ( ) I am not interested in quitting smoking

20. C07. Thinking about possible support options for your future attempts to quit smoking, which of the following might help you (choose all that apply)

Tick all that apply.

- [ ] Face-to-face counselling
- [ ] Access to an on campus mobile smoking cessation unit
- [ ] Telephone counselling
- [ ] Text messages
- [ ] Mobile phone app
- [ ] Online resources
- [ ] Medication
- [ ] Support group face-to-face
- [ ] Support group online
- [ ] Alternative treatments such as hypnotherapy or acupuncture

Other: [ ] ______________________

Section D - Challenges to Stopping Smoking

D00. The following statements refer to different challenges or problems associated with stopping smoking

D01. Please rate each of these factors in your most recent attempt to quit or thoughts about quitting smoking

21. Intrinsic factors

Mark only one oval per row.

	Not a challenge	Minor challenge	Moderate challenge	Major challenge
Withdrawal symptoms (e.g. depression, anxiety, restlessness, irritability, sleeplessness, craving, etc.)	◯	◯	◯	◯
Feeling lost without cigarettes	◯	◯	◯	◯
Being addicted to cigarettes	◯	◯	◯	◯
Having strong emotions or feelings such as anger, or feeling upset when I tried to stop smoking	◯	◯	◯	◯
Something stressful happened when I was trying to stop smoking	◯	◯	◯	◯
Thinking about never being able to smoke again after we stop smoking	◯	◯	◯	◯
Getting bored	◯	◯	◯	◯

Getting bored when I was trying to stop smoking				
Seeing things or people which reminded me of smoking	◯	◯	◯	◯
Easy availability of cigarettes	◯	◯	◯	◯

22. Extrinsic factors

Mark only one oval per row.

	Not a challenge	Minor challenge	Moderate challenge	Major challenge
Difficulty in finding someone to help me to stop smoking				
Lack of support or encouragement from health professionals to stop smoking				
The cost of stop-smoking medicines such as nicotine replacement therapy				
Fear of side effects from stop-smoking medications				
Lack of encouragement or help from family or friends to stop smoking				
Fear of weight gain if I stopped smoking				
Family members or				

Family members or friends encouraging me to smoke				
Fear of failing to stop smoking				
Belief that medicines to stop smoking do not work				
Fear that stopping smoking may interrupt social relationships				
Belief that I can stop smoking in the future if I need to				
Use of other substances like cannabis.				

Section E - Second hand smoke (think about both your and other people's smoke)

E00. I would like to ask you a few questions about smoking in different places

23. E01. Which of the following best describes the rules about smoking inside your family home

Mark only one oval.

- Smoking is acceptable inside your family home
- Smoking is generally not acceptable inside your home but there are exceptions
- Smoking is never acceptable inside of your home
- There are no rules about smoking in your home

24. E02. How often does anyone smoke inside of your family home

Mark only one oval.

- Daily
- Weekly
- Monthly
- Less often than monthly
- Never

25. E03. During the last 30 days, have you been aware of anyone smoking at any of these places (choose all that apply)

Tick all that apply.

- [ ] Indoors on university campus
- [ ] Outdoors on university campus
- [ ] Inside government buildings
- [ ] Inside healthcare facilities
- [ ] Inside restaurants or cafes
- [ ] Inside a cinema, theatre, music, sport or similar venue/event
- [ ] Outside a cinema, theatre, music, sport or similar venue/event
- [ ] On public transport
- [ ] In private transport

26. E04. Do you, or would you, support laws to prohibit smoking at any of these places

Mark only one oval per row.

	Yes	No	Don't know
Indoors on university campus	⬭	⬭	⬭
Outdoors on university campus	⬭	⬭	⬭
Inside government buildings	⬭	⬭	⬭
Inside healthcare facilities	⬭	⬭	⬭
Inside restaurants or cafes	⬭	⬭	⬭
Inside a cinema, theatre, music, sport or similar venue/event	⬭	⬭	⬭
Outside a cinema, theatre, music, sport or similar venue/event	⬭	⬭	⬭
On public transport	⬭	⬭	⬭

In private transport			

27. E05. Based on what you know or believe, which types of smoking can cause these illness (choose all that apply)

Tick all that apply.

	Stroke	Heart attack	Lung cancer	Other cancers	Chronic obstructive pulminary disease (COPD)
Factory made cigarettes	☐	☐	☐	☐	☐
Roll your own cigarettes with filters	☐	☐	☐	☐	☐
Roll your own cigrettes without filters	☐	☐	☐	☐	☐
Pipes full of tobacco	☐	☐	☐	☐	☐
Cigars, cheroots, cigarillos	☐	☐	☐	☐	☐
Waterpipe	☐	☐	☐	☐	☐
Electronic cigarettes	☐	☐	☐	☐	☐
Chewing tobacco	☐	☐	☐	☐	☐
Electronic hookah	☐	☐	☐	☐	☐

Section F - Media exposure

F00. The next question asks about your exposure to the media and advertisements in the last 30 days

28. F01. Where have you noticed information (choose all that apply)

Tick all that apply.

	Warning of dangers of use or encouraging quitting of tobacco products	Promoting tobacco smoking products	Promoting smokeless products
Newspapers or magazines (printed copies)	☐	☐	☐
Television	☐	☐	☐
Radio	☐	☐	☐
Billboards or posters	☐	☐	☐
In stores where tobacco products are sold	☐	☐	☐
Cinemas	☐	☐	☐
Public transportation vehicles or stations	☐	☐	☐
Sporting events or venues	☐	☐	☐
Internet (non-social media)	☐	☐	☐
Social media	☐	☐	☐

Cigarette packaging	☐	☐	☐

Section G - The impact of Covid 19

29. And finally, how has Covid 19 impacted your smoking habits and attitudes positively or negatively ?

Many thanks for your participation.

Your responses are all anonymised with no identifiable information collected in the survey. All responses will be analysed together and reported locally in poster format and at a university seminar with the potential for presentation at national and international health care conferences. A research paper will be submitted to an international health care journal. Your support in sharing your experiences, knowledge and attitudes towards smoking cessation are greatly appreciated. You will receive a single reminder email but do please only complete the survey once. Please click 'submit' to send your responses to the research team. You will then be offered the opportunity to claim 10 volunteer hours.

For further information contact:
Dr Mansour Tobaiqy
mtobaiqy@uj.edu.sa
Assistant Professor Pharmacology
Department
School of Medicine
University of Jeddah

30. If you wish to receive volunteer hours, please enter your name, the name of your department or college and your university identification number. This information will be held separately from your other answers so as to maintain your anonymity.

This content is neither created nor endorsed by Google.

Google Forms
